# Assessing the nonlinear association of environmental factors with antibiotic resistance genes (ARGs) in the Yangtze River Mouth, China

**DOI:** 10.1038/s41598-023-45973-9

**Published:** 2023-11-21

**Authors:** Jiazheng Miao, Yikai Ling, Xiaoyuan Chen, Siyuan Wu, Xinyue Liu, Shixin Xu, Sajid Umar, Benjamin D. Anderson

**Affiliations:** 1 Division of Natural and Applied Science, Duke Kunshan University, Kunshan, Jiangsu China; 2 Department of Biomedical Informatics, Harvard Medical School, Boston, MA USA; 3 Department of Epidemiology, Bloomberg School of Public Health, Johns Hopkins University, Baltimore, MD USA; 4 Department of Biomedical Engineering, Whiting School of Engineering, Johns Hopkins University, Baltimore, MD USA; 5 Department of Statistics, University of Michigan, Ann Arbor, MI USA; 6 Global Health Research Center, Duke Kunshan University, Kunshan, Jiangsu China; 7 Department of Environmental and Global Health, College of Public Health and Health Professions, and Emerging Pathogens Institute, University of Florida, Gainesville, FL 32610 USA

**Keywords:** Environmental sciences, Natural hazards

## Abstract

The emergence of antibacterial resistance (ABR) is an urgent and complex public health challenge worldwide. Antibiotic resistant genes (ARGs) are considered as a new pollutant by the WHO because of their wide distribution and emerging prevalence. The role of environmental factors in developing ARGs in bacterial populations is still poorly understood. Therefore, the relationship between environmental factors and bacteria should be explored to combat ABR and propose more tailored solutions in a specific region. Here, we collected and analyzed surface water samples from Yangtze Delta, China during 2021, and assessed the nonlinear association of environmental factors with ARGs through a sigmoid model. A high abundance of ARGs was detected. Amoxicillin, phosphorus (P), chromium (Cr), manganese (Mn), calcium (Ca), and strontium (Sr) were found to be strongly associated with ARGs and identified as potential key contributors to ARG detection. Our findings suggest that the suppression of ARGs may be achieved by decreasing the concentration of phosphorus in surface water. Additionally, Group 2A light metals (e.g., magnesium and calcium) may be candidates for the development of eco-friendly reagents for controlling antibiotic resistance in the future.

## Introduction

Antimicrobial resistance (AMR) refers to the process by which bacteria, parasites, viruses, and fungi evolve mechanisms that protect them from drugs^1^. Due to the increasing prevalence, health effects, and economic impact of AMR, the World Health Organization has characterized AMR as “a complex global public health challenge” (p. XIX) that requires urgent action^1^. Countermeasures against antibacterial resistance (ABR) are of particular urgency because of severe inadequacy of alternative therapeutic options^1^. In 2019, it was estimated that 4.95 million deaths were associated with ABR, and 1.27 million deaths were attributable to ABR^2^. In a recent study that estimateed the economic burden of ABR in China, 27.45% of inpatient bacterial infections were antibiotic resistant, which is estimated to have caused a 1.5% increase in inpatient mortality rates and cost $77 billion in related societal expenses in 2017^3^. Moreover, bacteria are capable of spreading ABR at a high rate through the exchange of antibiotic resistant genes (ARGs) by horizontal gene transfer (HGT)^4^. ARGs are a group of genes that encode the proteins responsible for antibiotic resistance and are frequently carried on mobile genetic elements (MGEs) like plasmids, transposons, and integrons^4^. Through HGT, one bacterial cell transfers its MGEs to other bacterial cells, facilitating environmental dispersal of the ARGs^4^. Thus, ARGs have been recognized as an emerging environmental pollutant that poses a threat to human health^4^.

Earlier studies have investigated whether ABR would decrease when antibiotic use was reduced, but the results have indicated that ABR may persist due to the low cost of resistance, other biological functions of ARGs, selection at sub-minimum inhibitory concentration levesl, or compensatory evolution^5–7^. Later studies have further identified additional non-antibiotic substances as significant contributors to the propagation of ARGs^8^.

First, heavy metals are reported to have a co-selection effect on ARGs^9–11^. Because bacteria resist antibiotics and heavy metals through similar strategies (e.g., reduction in permeability, efflux, sequestration, etc.), heavy metals may also pose a selective pressure on ARGs. Furthermore, antibiotic resistant communities in urban lakes have also been identified to be more competitive than non-resistant communities at higher temperatures, but less competitive at higher concentrations of magnesium or salinity^12^. Second, microplastics (MPs) have also garnered attention for their role in enriching ARGs. Zhang et al.^13^ reported that MPs can directly absorb ARGs onto their surfaces within matriculate systems, thereby creating an ideal platform for microbial colonization. Furthermore, the presence of MPs has been associated with an increased potential for HGT, promoting the propagation of ARGs in municipal wastewater, as noted by Cheng et al.^14^. In addition to MPs, various common disinfectants, such as chlorination, and the disinfection by-products they generate have been shown to exert dose-dependent effects on ARG migration by influencing the permeability of cell membranes, as demonstrated by Guo, Yuan, and Yang^15^. Notably, low-intensity ultrasound has also been observed to enhance the efficiency of resistant gene transfer by augmenting cell membrane permeability, as documented by Song et al.^16^. These findings collectively highlight the diverse ways in which non-antibiotic materials can contribute to the dissemination of ARGs, shedding light on the intricate interactions between environmental factors and antibiotic resistance. Therefore, to effectively understand and mitigate ABR in a specific region, local environmental conditions must be investigated.

ARGs are mainly encapsulated in mobile genetic elements (MGE), such as plasmids^17^. Plasmids are genetic materials that are physically separate from chromosomes and replicate independently^18^. Due to these features, this study employed a plasmid-centric framework^19^ to study the effect of environmental factors on ARG abundance. Specifically, this study treated the plasmids as the unit of population dynamics and assumed the quantity followed logistic growth. This permits the assessment of nonlinear association between ARGs and environmental factors through a sigmoid model.

The Yangtze River Mouth is one of the most economically developed regions in China, which includes Shanghai City, the southern part of Jiangsu Province, and the northern part of Zhejiang Province^20^. Shanghai represents the primary geographical majority of the region with a high population density of 3823 persons/square kilometre as of 2018^21^. The quality of drinking water in this area (e.g., Yangcheng Lake in Suzhou and Huangpu River in Shanghai) is critical given that it is the primary water source for residents. Due to its economic and environmental importance, previous studies have investigated the prevalence of ARGs and patterns of antibiotic use in the area.

In 2020, 64 out of 69 types of antibiotics monitored in the surface water in China were detected in Shanghai with concentrations ranging from a few pg/L to hundreds of ng/L^22–24^. While  these studies  demonstrated the widespread distribution and persistence of antibiotics in this region, the environmental risk for most types of antibiotics were low^23,25^  based upon the calculated hazard quotients^26^. However, the environmental risks of enrofloxacin, amoxicillin, and oxytetracyclines to aquatic organisms, represented by the risk quotient method^27,28^, are distinctly higher than those in other countries^23^. As for ARGs, tetracycline resistance genes (*tet*), sulfonamide resistance genes (*sul*), and β-lactam were detected in waterbodies in the Yangtze Delta and surrounding regions^29,30^. For *sul* and *tet* genes, the detection frequency of *sul*I, *sul*II, *tet*A, *tet*G, *tet*M and *tet*O were markedly higher than *sul*III, which was still significantly higher than *tet*C^30^. It was noted that samples with more *sul* genes were more likely to contain higher levels of total sulfonamides, which was similarly observed for *tet* genes^29^.

In this study, we tested surface water samples for ARGs, antibiotics, and dissolved elements to better understand what environmental factors might contribute to the prevalence of ARGs in main water bodies of the Yangtze Delta.

## Methods

### Sampling sites and sample collection

In 2021, surface water samples were collected from sampling sites located along a watershed starting at Yangcheng Lake in Suzhou, running along the Wusong River, passing Dianshan Lake in Shanghai, running along the Huangpu River in Shanghai, and ending at an estuary at the Shanghai coastal area. The watershed connects most of the main water bodies in Suzhou and Shanghai. The sampling area contained four subregions: Dianshan Lake (Qingpu District, Shanghai), Huangpu River (Songjiang District, Shanghai), estuary (Jinshan District, Shanghai), and Kunshan (Suzhou).

Surface water samples were collected using brown HDPE bottles (NINGKE, China) that were dipped into the top 10 cm of the water body with the goal of collecting 1000 mL of water. Water temperature, salinity, and pH of the collected samples were immediately measured and recorded using a salinity and pH meter (BANTE, China) according to the manufacturer’s instructions. Sample bottles were transported on wet ice within 3 h to Duke Kunshan University where 650 mL of each sample was filtered through a 0.45 µm glass microfiber filter membrane (Titan, China). The filtrate and filter membranes were stored in a − 80 °C freezer.

### Measurement of ARGs

Material captured by the filter membrane was reconstituted in 10 mL of ultrapure water. A TaKaRa MiniBEST Bacteria Genomic DNA Extraction Kit was used to extract 240 µL of DNA from 10 mL of stored sample, according to the manufacturer’s instructions. Quantitative PCR was used to screen extracts for ARGs using the SYBR Green Real-Time PCR assay (TaKaRa Co. Dalian, China) on a Mic qPCR Cycler (BioMolecular Systems, EI Cajon, CA), according to the manufacturer’s instructions. One sample was randomly selected from each of the four subregions. A total of 26 primers for ARGs were used to screen the four samples. The ARGs include 15 tetracycline resistance genes (*tetA*, *tetC*, *tetE*, *tetK*, *tetL*, *tetA/P*, *tetG*, *tetM*, *tetO*, *tetQ*, *tetS*, *tetT*, *tetW*, *tetBP*, and *tetX*), 4 β-lactam resistance genes (*bla*_CTX-M_, *bla*_TEM_, *bla*_SHV_, and *bla*_ampC_), 3 sulfonamide resistance genes (*sul*I, *sul*II, and *sul*III), 3 macrolides resistance genes (*ereA*, *ereB*, and *mphA*), and 1 beta-lactam resistance gene (*ampr*). Primer sequences and cycling conditions for the quantitative PCR experiments are listed in Table S1. Cultured environmental samples were used as positive controls.

Based on the ARG screening results (Fig. S1a,b), the three most abundant ARGs were selected for further quantitative PCR experiments: *tetBP*, *ampr*, and *tetO*. Each gene was commercially synthesized and used as an internal standard to determine absolute abundance.

### Measurement of dissolved elements concentrations

After filtration, approximately 150 mL of stored filtrate was acidified to pH < 2 by (1 + 1) nitric acid and preserved in a − 80 °C freezer. The samples were sent to the Nanjing Institute of Geography & Limnology, Chinese Academy of Sciences and measured for calcium (Ca), potassium (K), magnesium (Mg), sodium (Na), silicon (Si), silver (Ag), mecury (Hg), thallium (Tl), aluminium (Al), arsenic (As), boron (B), barium (Ba), beryllium (Be), cadmium (Cd), cobalt (Co), chromium (Cr), copper (Cu), iron (Fe), manganese (Mn), molybdenum (Mo), nickel (Ni), phosphorus (P), lead (Pb), antimony (Sb), selenium (Se), tin (Sn), strontium (Sr), titanium (Ti), vanadium (V), and zinc (Zn) by ICP-MS 7900 (Agilent, US), as described in Ref.^31^.

### Measurement of antibiotic concentration

Concentrations of amoxicillin and tetracycline were measured using 500 mL of stored filtrate using the Amoxicillin ELISA kit (Shenzhen Lvshiyuan Biotechnology Co., Ltd, China) and Tetracycline ELISA kit (Shenzhen Lvshiyuan Biotechnology Co., Ltd, China), according to the manufacturer’s instructions. The procedures of the two kits were technically the same. Briefly, samples and standard solutions with known antigen (amoxicillin/tetracycline) concentration were separately mixed with corresponding enzyme conjugates (enzyme-labelled antibodies) on a micro-well plate where micro-well strips were pre-coated with coupling antigens. The antigens in the sample and the coupling antigens would compete for the enzyme conjugates. After removing the mixed solution and adding TMB substrate for coloration, the enzyme conjugates bound with sample antigens were removed and only those bound with pre-coated antigens remained, which led to higher optical density (OD) values in the samples with lower antigen concentrations. The OD values of the solutions generated from samples and standard solutions were measured at a wavelength of 450 nm. All procedures above were performed in duplicate for each sample and standard solution. A standard curve of absorption versus antigen concentration was generated with the OD values from the standard solutions, and the OD values from the samples were compared to the standard curve to get the antigen concentration.

### Environmental risk assessment of antibiotics

To assess the potential risk of antibiotics in the environment, a hazard quotient (HQ) was used, which is the ratio between the measured environmental concentration (MEC) of the antibiotic and the predicted no-effect concentration (PNEC)^26^. HQ values > 1.0 indicate a potential risk to the aquatic system^32^. This study adopts PNEC values proposed by Bengtsson-Palme and Larsson, 2016 (0.25 μg/L for amoxicillin and 1 μg/L for tetracycline)^33^.

### Mathematical modeling

This study treated the plasmids as the unit of logistic population growth and assumed that ARG abundance is proportional to the quantity of plasmids. The fitness cost of conveying plasmids and environmental pressures were represented as coefficients on growth rate, as in Ref.^34^. Therefore, the influence of environmental factors can be described as:

1 $$\frac{dP}{dt}={g}_{0}\left(\rho -c\right)P\left(1-\frac{P}{K}\right),$$

2 $$\rho =\mu M,$$

3 $$N=P\varepsilon ,$$

where $P$ is the population size of the MGE, $t$ is the time, $K$ is the maximum population that the environment could support, ${g}_{0}$ is the growth rate in ideal conditions, $\rho$ is the death penalty caused by environmental chemicals, and $c$ is the energy cost to convey the plasmid. The death penalty $\rho$ is proportional to the chemical concentration $M$, while $\mu$ is a coefficient. $N$ is the total number of ARGs, which is equal to the product of $P$ and $\varepsilon$, the average number of ARG per plasmid.

As described by the Eqs. (1)–(3), the heavier the environmental pressure is, the more likely the plasmids will replicate. However, if the environmental risk does not overcome the energy cost, the bacteria will start to degrade the plasmids. Deducing from the Eqs. (1)–(3), the total copy number of ARGs could be expressed as a function of time $t$:

4 $$N\left(t\right)=\frac{K\varepsilon }{1+(\frac{K\varepsilon -{N}_{0}}{{N}_{0}}){e}^{{-g}_{0}(\mu M-c)t}},$$

where ${N}_{0}$ is the initial copy number of ARGs. Because all the samples were collected intensively with limited time variance, the data could be assumed as being collected simultaneously at ${t}_{1}$, such that:

5 $$N\left({t}_{1}\right)=\frac{K\varepsilon }{1+(\frac{K\varepsilon -{N}_{0}}{{N}_{0}}){e}^{{-(g}_{0}{t}_{1}\mu M+{g}_{0}{t}_{1}c)}}.$$

If we substitute ${g}_{0}{t}_{1}\mu$ as $w$, and ${g}_{0}{t}_{1}c-$ as $b$ in the Eq. (5):

6 $$N\left({t}_{1}\right)=\frac{K\varepsilon }{1+{e}^{-(wM+b)}}\propto sigmoid\left(wM+b\right).$$

This Eq. (6) allows for using logistic regression to find the optimal $w$ and $b$, and represents the nonlinear association of ARGs and environmental factors as the correlation between $N$ and $sigmoid(wM+b)$.

### Statistical method

ARG screening data was calculated to relative abundance using the median Cq value of *tetE* in Sample S7 as a reference with the following formula:

$${\text{Relative}}\;{\text{abundance}} = 2^{{({\text{Cq}}_{{{\text{reference}}}} - {\text{Cq}})}} .$$

*TetBP*, *ampr*, and *tetO* qPCR data was calculated to absolute abundance using a standard curve derived by serially diluting (tenfold) synthetic genes at a range of 10^12^ copies/mL to 10^2^ copies/mL. An ANOVA test was performed by the *aov* function in R base to explore the regional difference in ARG abundance. The samples also underwent scaled hierarchical clustering with Euclidean distance and complete linkage through the *scale*, *dist*, and *hclust* functions in R base.

**Figure 1:**
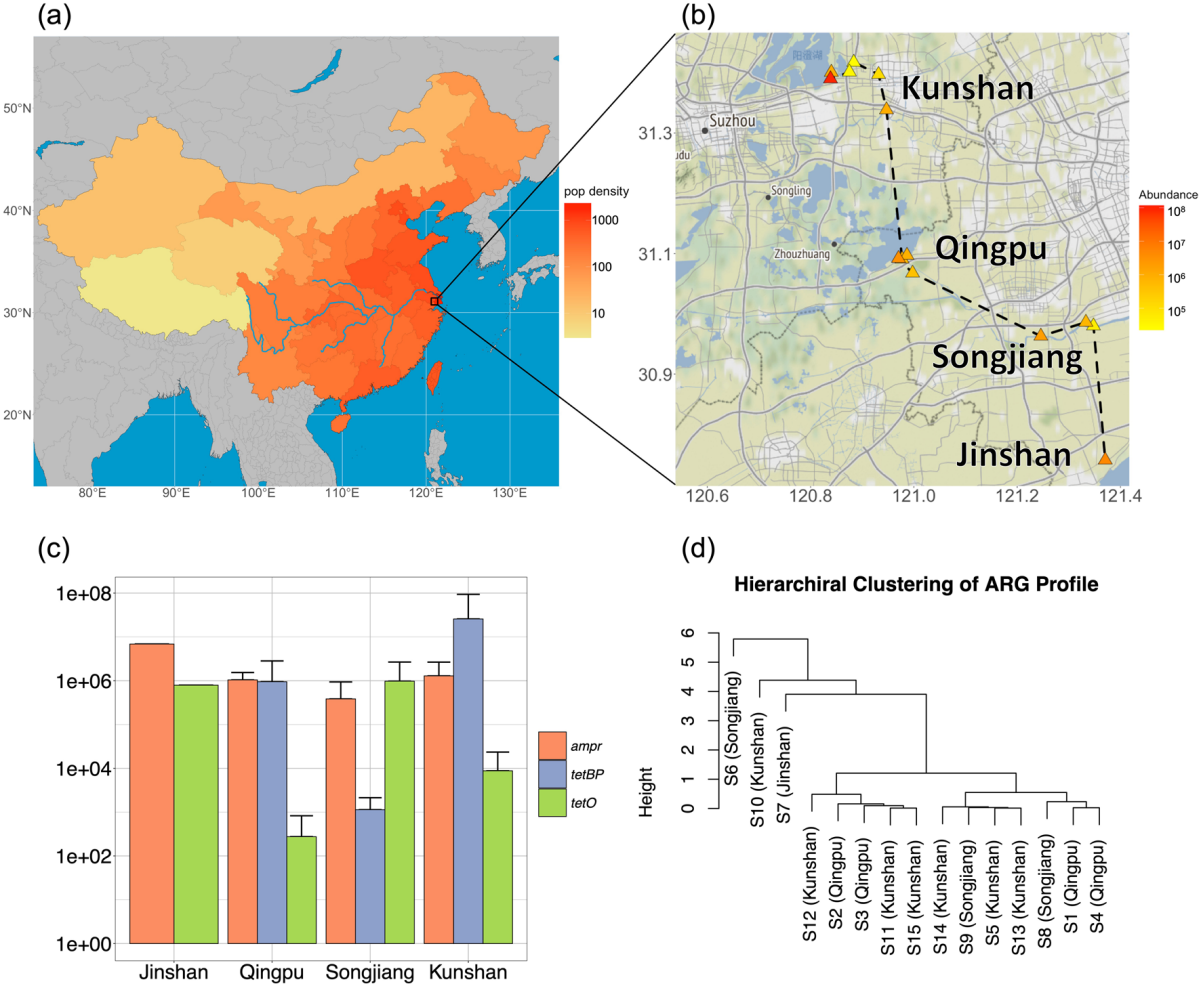
Spatial distribution and the profile of ARGs in the sampling area. (**a**) Map of China with population density in 2020. Unit of population density: persons/square kilometre. The blue line represents the Yangtze River. (**b**) Spatial distribution of samples and absolute abundance of total ARGs in each sample. Unit of total ARG abundance: copies/mL. The black dash represents the sampling lane. (**c**) Mean absolute abundance of ARGs in the four sampling regions. Unit of ARG abundance: copies/mL. (**d**) Hierarchical clustering of the samples based on the ARG abundance.

All figures were generated using R (version 4.0 and 4.1). Figure 1a used country border data and river data from the rnaturalearth package. Population density data was collected by the Ministry of Civil Affairs of the People’s Republic of China^35^ and Statistical Bureau of Republic of China^36^. Figure 1b adopted the Stamen map through the ggmap package.

To assess the nonlinear association between ARG abundance and environmental factors, as described in Eq. (6), the variables were first normalized through max–min scaling and underwent a pairwise logistic regression using the *glm* function in the base package of R. The nonlinear association was represented as the Pearson correlation coefficient between the true ARG abundance and the predicted abundance by the glm model, calculated by the *cor* function in R base.

## Results

The ARG screening results were shown in Fig. S1. Figure S1a shows the sum of relative abundance of the four screening samples. The three most abundant ARGs were *tetBP*, *ampr*, and *tetO*. The *ereA*, *ereB*, and *mphA* ARGs were not detected in the samples. Therefore, *tetBP*, *ampr*, and *tetO* were selected for further study*.*

The spatial distribution of the samples and absolute abundance of total ARGs (sum of *tetBP*, *ampr*, and *tetO*) are shown in Fig. 1b. The Kunshan sample had a mean copy number of 1.3e+06 copies/mL for *ampr* (SD = 1.4e+06), 2.6e+07 copies/mL for *tetBP* (SD = 6.8e+07), and 8.8e+03 copies/mL for *tetO* (SD = 1.5e+04). The mean copy number at Qingpu was 1.0e+06 copies/mL (SD = 4.9e+05) for *ampr*, 9.5e+05 copies/mL (SD = 1.9e+06) for *tetBP*, and 2.8e+02 copies/mL (SD = 5.5e+02) for *tetO*. For Songjiang, *ampr* had a mean abundance of 3.8e+05 copies/mL (SD = 5.5e+05), *tetBP* averaged 1.1e+03 copies/mL (SD = 1.0e+03), and *tetO* 9.8e+05 copies/mL (SD = 1.7e+06). Finally, for Jinshan, *tetBP* was not detected, while *ampr* had 6.9e+06 copies/mL and *tetO* had 7.9e+05 copies/mL. Notably, an ARG hotspot (S10) was found in Yangcheng Lake, Kunshan, which contained an elevated amount of *tetBP* (Fig. S1b). However, no clear regional pattern was observed. The mean absolute abundance and composition of ARGs in the four regions are shown in Fig. 1c. The ARG *ampr* accounted for the majority of detected concentrations in all the regions. However, all the results showed a large standard deviation, indicating high variability within each region. The ANOVA test returned a p-value of 0.798, indicating no regional differences. Finally, consistent with the ANOVA test results, Fig. 1d shows that the hierarchical clustering of the samples, based upon ARG abundance, does not place samples from the same region in closer branches.

As for the concentration of dissolved elements, the complete measurement results are shown in the Supplementary Data. The range of concentration for light metals is the highest among the tested elements, which is 5.90–61.3 mg/L for K, 15.3–48.2 mg/L for Na, 21.3–35.0 mg/L for Ca, 5.59–8.62 mg/L for Mg, 83.5–645 μg/L for Al, and 0–0.091 μg/L for Be. The range for non-metal elements, sorted in decreasing order, is 0–0.462 mg/L for Si, 24.3–459 μg/L for P, 99.2–139 μg/L for B, 2.22–5.01 μg/L for As, and 0–0.535 μg/L for Se. Finally, the range for heavy metals, sorted in decreasing order, is 50.7–862 μg/L for Fe, 110–257 μg/L for Sr, 15.5–207 μg/L for Mn, 0–45.9 μg/L for Ba, 0–34.5 μg/L for Ag, 0.339–11.6 μg/L for Ti, 0–11.1 μg/L for Zn, 2.46–6.18 μg/L for Mo, 0.860–5.85 μg/L for V, 0.553–4.42 μg/L for Ni, 0.333–2.92 μg/L for Pb, 0–2.19 μg/L for Sn, 0–2.08 μg/L for Cr, 0–2.07 μg/L for Cu, 0.617–1.57 μg/L for Sb, 0.070–0.643 μg/L for Co, 0–0.047 μg/L for Hg, 0–0.031 μg/L for Cd, and 0 μg/L for Tl. In general, the water quality was good for all of the tested samples (Table 1) as classified by the Environmental Quality Standard for Surface Water of People’s Republic of China^37^, with one exception. Specifically, Sample S8 collected from a drinking water reserve in Songjiang District had an iron concentration of 417.436 μg/L and phosphorus concentration of 237.872 μg/L that exceeds the standard limit for drinking water (300 μg/L for iron and 200 μg/L for phosphorus), as shown in Table 2.

**Table 1:** Water quality rank (according to Environmental Quality Standard for Surface Water of PRC^37^) and concentration (μg/L) of dissolved elements.

Sample	Area	P		Cu		Zn		As		Se		Hg		Cd		Pb		Cr	
S1	Qingpu	III	128.006	I	0.174	I	ND	I	4.386	I	ND	I	0.047	I	0.018	I	2.922	I	0.973
S2	Qingpu	III	136.442	I	0.427	I	ND	I	4.345	I	ND	I	0.025	I	0.012	I	0.791	I	1.28
S3	Qingpu	II	94.828	I	0.433	I	7.425	I	4.288	I	ND	I	0.021	I	0.026	I	1.372	I	0.742
S4	Qingpu	II	79.516	I	0.056	I	1.762	I	5.011	I	ND	I	0.034	I	0.021	I	2.112	I	0.752
S5	Kunshan	II	37.645	I	ND	I	ND	I	2.329	I	ND	I	ND	I	0.017	I	1.914	I	0.522
S7	Jinshan	V	458.891	I	ND	I	5.135	I	3.743	I	ND	I	ND	I	0.004	I	0.668	I	2.074
S8*	Songjiang	IV	237.872	I	ND	I	2.272	I	3.626	I	0.535	I	ND	I	0.011	I	0.831	I	0.6
S9	Songjiang	III	180.972	I	0.529	I	3.414	I	3.276	I	ND	I	ND	I	0.023	I	1.205	I	1.33
S10	Kunshan	II	34.28	I	ND	I	ND	I	2.359	I	ND	I	ND	I	ND	I	0.457	I	ND
S11	Kunshan	II	30.058	I	ND	I	ND	I	2.442	I	ND	I	ND	I	0.005	I	0.642	I	ND
S12*	Kunshan	II	45.482	I	ND	I	9.226	I	2.708	I	ND	I	ND	I	ND	I	0.332	I	0.264
S13*	Kunshan	II	24.372	I	ND	I	ND	I	2.217	I	ND	I	ND	I	0.004	I	0.646	I	0.113
S14	Kunshan	IV	258.324	I	0.275	I	0.72	I	2.712	I	ND	I	ND	I	0.022	I	0.967	I	0.351
S15	Kunshan	III	114.272	I	2.07	I	11.143	I	2.309	I	ND	I	ND	I	0.031	I	2.364	I	1.125

Rank I is applicable for national nature reserves. Rank II is for first-class drinking water reserves. Rank III is for second-class drinking water reserves. Rank IV is for industry water and non-exposure entertainment water sources. Rank V is for agricultural water sources.

*ND* not detected.

*Labelled samples are from drinking water reserves.

**Table 2:** Additional requirements from environmental quality standard for surface water of PRC for drinking water reserves.

Sample	Area	Mo		Co		Be		B		Sb		Ni		Ba		Ti		V		Tl		Fe	
S8	Songjiang	W	3.538	W	0.326	W	ND	W	99.268	W	0.751	W	0.548	W	ND	W	6.237	W	2.632	W	ND	**E**	**417.436**
S12	Kunshan	W	3.302	W	0.153	W	ND	W	119.726	W	1.407	W	1.998	W	ND	W	1.985	W	2.021	W	ND	W	153.924
S13	Kunshan	W	2.453	W	0.075	W	ND	W	115.066	W	0.742	W	1.312	W	ND	W	0.596	W	0.856	W	ND	W	61.913

*W* concentration is within the limit for drinking water reserves, *E* means exceeding the requirement, *ND* not detected.

Values that exceed the corresponding risk thesholds are in bold.

Measured antibiotic concentrations are shown in Table 3. Amoxicillin and tetracycline were detected in all of the samples. For tetracycline, the concentration ranged from 110 to 190 ng/L, with the maximum concentration found at Jinshan District and the minimum at Qingpu. Figure S2 shows the comparison of these results with previous studies of tetracycline concentration in the surface water of Shanghai^22,38,39^. The total tetracycline concentration is the weighted sum of tetracycline derivatives, by the cross-reactivity provided by the manufacturer, as listed in Table S2. The results of this study are consistent with those previous studies, which show that the concentration of total tetracycline is lower than 200 ng/L over the past decade. Calculated from the PNEC of tetracycline (1 μg/L or 1000 ng/L), the HQ value of tetracycline is lower than 1 (MAX = 0.19), indicating no risk to the aquatic environment. As for amoxicillin, the concentration rangeed from 210 to 560 ng/L, with the maximum concentration found at Jinshan again. The HQ values ranged from 0.84 to 2.24. Jinshan, Kunshan, and Songjiang all had environmental sites with elevated risk, with Jinshan being the highest. However, the high concentration of amoxicillin, or beta-lactam, have not been reported in previous studies, which appears to be a new finding.

**Table 3:** Concentrations (unit: ng/L) and HQ of amoxicillin and tetracycline.

Sample	Area	Amoxicillin conc. (HQ)	Tetracycline conc. (HQ)
S1	Qingpu	210 (0.84)	110 (0.11)
S2	Qingpu	240 (0.96)	110 (0.11)
S3	Qingpu	230 (0.92)	130 (0.13)
S4	Qingpu	280 (0.92)	120 (0.12)
S5	Kunshan	**280 (1.12)**	120 (0.12)
S6	Songjiang	**270 (1.08)**	140 (0.14)
S7	Jinshan	**560 (2.24)**	190 (0.19)
S8	Songjiang	230 (0.92)	130 (0.13)
S9	Songjiang	230 (0.92)	120 (0.12)
S10	Kunshan	**270 (1.08)**	120 (0.12)
S11	Kunshan	250 (1.00)	130 (0.13)
S12	Kunshan	250 (1.00)	120 (0.12)
S13	Kunshan	**260 (1.04)**	130 (0.13)
S14	Kunshan	**310 (1.24)**	150 (0.15)
S15	Kunshan	**310 (1.24)**	150 (0.15)

Detection limit: 100 ng/L.

*HQ* hazard quotient.

Values that exceed the corresponding risk thesholds are in bold.

**Figure 2:**
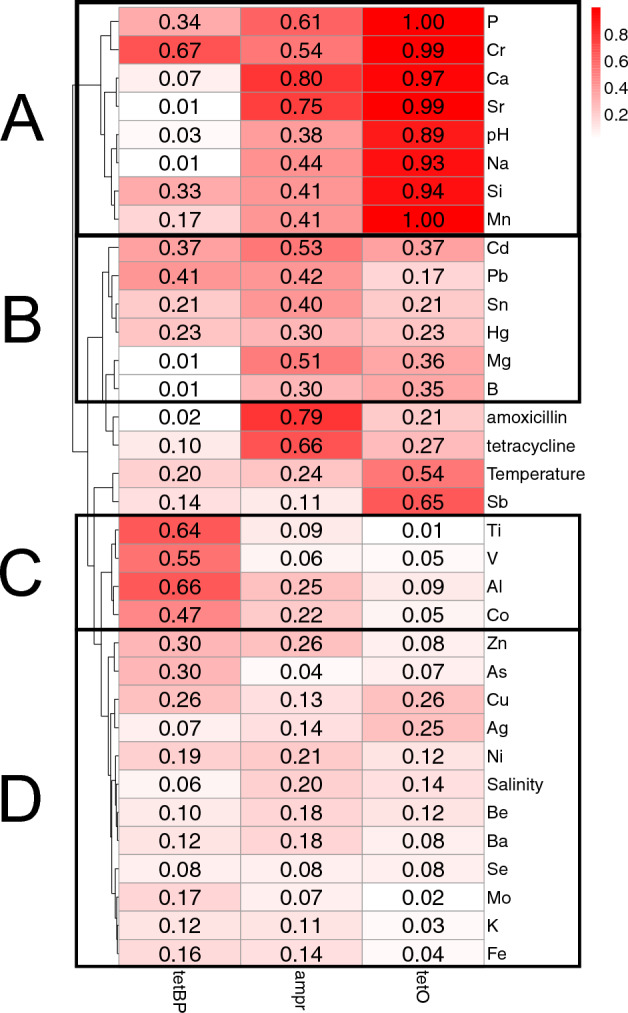
Heatmap of nonlinear association of ARG concentrations and environmental parameters.

Figure 2 shows the nonlinear association of ARGs with various environmental factors, including antibiotics, salinity, temperature, and dissolved elements. Amoxicillin shows a strong association with *ampr* (r = 0.79), while tetracycline has a limited association with *tetO* or *tetBP* (r = 0.27, 0.1). For physical water properties, salinity has negligible association with *tetBP* (r = 0.06), *ampr* (r = 0.19), and *tetO* (r = 0.14), and water temperature also shows negligible association with *tetBP* and *ampr* (r = 0.2, r = 0.22), but moderate association with *tetO* (r = 0.54).

As for the dissolved elements, the variables were grouped into 4 clusters based on the Manhattan distance (sum of absolute difference across all dimensions) of association coefficients. Cluster A, C, and D have distinct characteristics from each other. First, Cluster A includes the most associative elements, which are pH, Na, Ca, Sr, Cr, P, Si, and Mn. All of these elements have a strong association with *tetO* (r ≥ 0.89). In addition, Ca and Sr have strong associations with *ampr* as well (r = 0.79, 0.74). Second, Cluster C, including Ti, V, Al, and Co, show weak associations with *tetBP* (0.47 ≤ r ≤ 0.66), and have negligible associations with *tetO* or *ampr* (r ≤ 0.24). This cluster is the most associative group of *tetBP*. Third, the elements in Cluster D (Zn, As, Cu, Ag, Ni, Ba, Be, Se, Mo, K, and Fe) and Hg in Cluster B have negligible associations with all three ARGs (r ≤ 0.3). Fourth, the remaining elements in Cluster B (Cd, Pb, Sn, Mg, and B) show relatively equal associations to all three ARGs. While Cd and Mg have moderate associations with *ampr* (r = 0.53, 0.50), and Mg and B have negligible associations with tetBP, the other associations are all relatively low (0.2 < r < 0.5). Last, Sb, as an outgroup in the hierarchical clustering, has a moderate association with *tetO* (r = 0.65), and negligible associations with *tetBP* and *ampr* (r = 0.14, 0.09).

**Figure 3:**
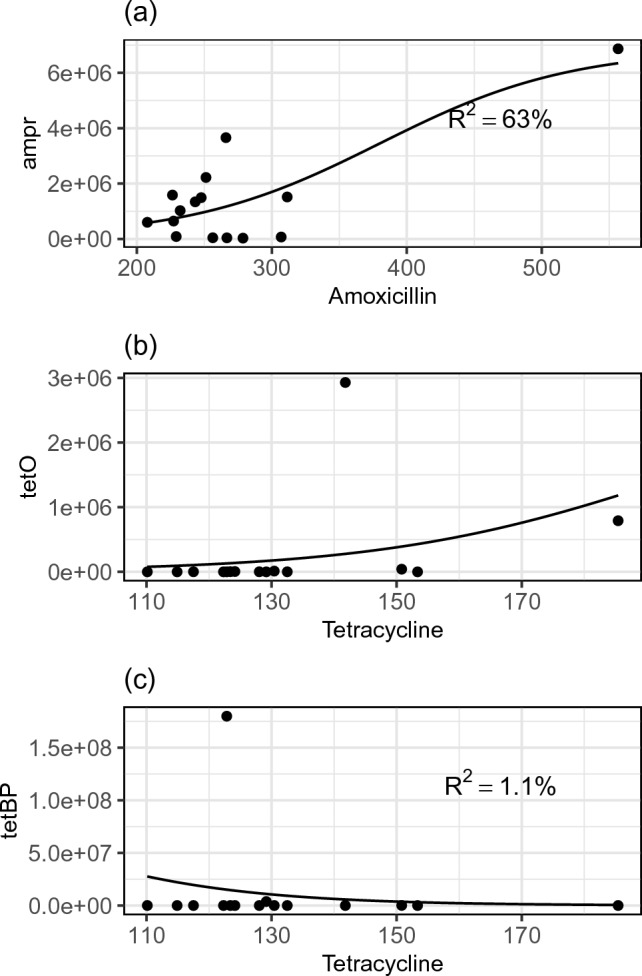
Relationship of antibiotics with ARGs. The black line is fitted through the sigmoid model. (**a**) Amoxicillin vs. *ampr*. (**b**) Tetracycline vs. *tetBP*. (**c**) Tetracycline vs. *tetO*. Unit: ampr (copies/mL), tetO (copies/mL), amoxicillin (ng/L), tetracycline (ng/L).

**Figure 4:**
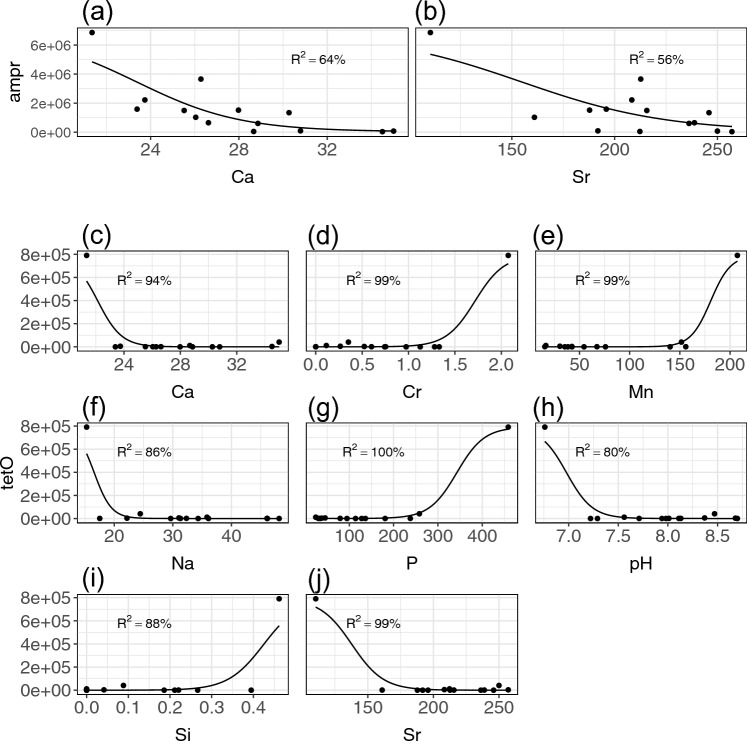
Relationship of most associative elements with ARGs. (**a**) Ca vs. *ampr*. (**b**) Sr vs. *ampr*. (**c**) Ca vs. *tetO*. (**d**) Cr vs. vs. *tetO*. (**e**) Mn vs. *tetO*. (**f**) Na vs. *tetO*. (**g**) P vs. *tetO*. (**h**) pH vs. *tetO*. (**i**) Si vs. *tetO*. (**j**) Sr vs. *tetO*. The black line is fitted through the sigmoid model. Unit: ampr (copies/mL), tetO (copies/mL), Ca, Si, Na (mg/L), Cr, Mn, P, Sr (μg/L).

Based on their associations, environmental factors were selected and the sigmoid relationship with ARGs and other assessed factors were visualized. Figure 3 shows the results of predicting the ARGs from corresponding antibiotics using the sigmoid model. It can be determined that amoxicillin has a significant association with *ampr*, while tetracycline is not associated with *tet* genes. Figure 4 shows the results of predicting the *tetO* and *ampr* from the most associative variables (r > 0.7) using the sigmoid model. The predicted sigmoid curves fit the ARG abundance well.

## Discussion

This study aimed to assess the nonlinear association of environmental factors and the prevalence of ARGs in main water bodies in the Yangtze River Mouth area. In summary, a high abundance of ARGs were detected and four categories of environmental factors were found to be associated with ARG abundance.

First, as shown in Figs. 1 and 2, a high abundance of ARGs were detected. Compared with historical data of Shanghai collected in July 2013^29^, the ARG abundance in Huangpu River detected in this study was five magnitudes higher. Specifically, historical data shows that the abundance of *tetO* was at a magnitude of 10 copies/mL, and other tetracycline-resistant genes were at a magnitude of 10 to 10^4^ copies/mL^29^. But in this study, *tetO* abundance in Shanghai reached up to 10^6^ copies/mL (Fig. 2). Additionally, the hotspot sample from Yangcheng Lake, Kunshan, contained *tetBP* at a magnitude of 10^8^ copies/mL (Fig. 1). This finding suggests there could have been an increase of antibiotic resistant bacteria in the Yangtze Reiver Mouth area during the past decade. What is more, the hotspot and large standard deviations of ARG concentrations imply that there might be point sources of ARG pollution, which elevate ARG concentrations locally (e.g., hotspot of Sample S10), but may not disperse over a wide area.

Given the high abundance of ARGs, the first potential contributor is believed to be antibiotics, especially amoxicillin. As shown in Table 3, the HQ of amoxicillin exceeded 1 in all regions except Qingpu. This result is in line with the sigmoid nonlinear association, which indicates ampr is strongly related with amoxicillin. However, high concentrations of amoxicillin have not been reported in the Yangtze River Mouth area by previous studies. 

However, the high concentration of amoxicillin alone is  not able to explain all ARG results. Specifically, in contrast to amoxicillin, tetracycline persistence in the environment was limited, with ELISA results showing that all samples contained less than 200 ng/L and the risk is far lower than the threshold (Table 3). This finding is consistent with other studies demonstrating similar tetracycline antibiotic concentrations in surface water in Shanghai at a magnitude of ng/L^22–24^. However, despite low antibiotic risk, *tet* genes remain persistent in the aquatic system. This result is consistent with the sigmoid modeling results that tetracycline is not associated with *tet* genes. Notably, such persistence of tetracycline resistance was also reported in coastal regions of Southeast China, which is geologically close to this study’s sampling region^40^. These findings suggest that other factors were driving the persistence of *tet* genes.

In this case, we investigated the top associative environmental factors with *tet* genes, and identified phosphorus and co-selection by heavy metals as the second and third potential driving forces of ARG abundance. As shown in Figs. 2 and 4g, phosphorus exhibits a strong positive association with *tetO*. One possible explanation for this association is that phosphorus, as an essential biological nonmetal element, enriches bacterial growth in freshwater^41,42^. The abundance of ARGs proliferates as the quantity of bacteria increases, which has also been posited by other studies^43,44^. This positive association between phosphorus and ARGs is also reported by other studies in eutrophic environments^45,46^. Though additional assessment is needed to better understand the relationship between phosphorous concentrations and ARG, the findings seem to suggest that regulating phosphorous eutrophication could be a practical approach to managing ARG development in surface water, as proposed by Wang et al.^46^.

In terms of heavy metals, as shown in Fig. 4, Cr and Mn exhibit a strong positive association with *tetO*. The positive association of chromium could be a result of co-selection between tetracycline-resistance genes and chromium-resistance genes through co-resistance mechanisms. Co-resistance describes the spatial association of HMRGs and ARGs when they are located on the same genetic elements, such as plasmids and transposons. This association means either heavy metals or antibiotics can be the selection pressure for both resistance genes. A previous study cured plasmids from *Salmonella abortus* equi strains and found resistance genes for ampicillin, with As, Cr, Cd and Hg encapsulated simultaneously in the plasmids^47^. However, co-selection of Mn and ARGs is rarely reported. Further research is needed to confirm whether Mn truly has co-selective pressure on ARGs.

What is more, through investigating the nonlinear association between ARGs and other soluble cations, this study identified Group 2A light metals as potential suppressors of ARGs. First, both Ca and Sr were found to have strong negative associations with *ampr* and *tetO* (Fig. 4). Interestingly, as noted, a previous study reported that magnesium (Mg) could suppress resistant bacterial communities^12^. Mg, Ca, and Sr all belong to Group 2A on the periodic table. This means that the three elements have similar chemical properties, so it is possible that the three elements have a potential inhibition effect through similar mechanisms.

One potential explanation is that these light metals can all place selective pressure on bacteria  with or without resistance genes. First, Sr has been experimentally proven to have cytotoxicity on bacteria. The cytotoxicity is achieved through induction of oxidative radicals that damage the cell membrane, or inactivation of adenosine triphosphate (ATP) synthesis^48,49^. What is more, molecular biology studies have also reported selective inhibition effects of Ca on antibiotic-resistant bacteria. Ca may be involved directly in the destabilization of the *Staphylococcus aureus* cell membrane, by forming a complex with Cardiolipin (CL), one major component of the bacterial cell membrane, thus destabilizing the cell membrane and eventually causing cell death^50^. The disrupted cell membrane partly loses the barrier function, which can cause leakage of some cell contents and finally lead to cell death^50^. In addition, Ca could suppress bacterial growth through inhibiting peptidoglycan-layer (PG-layer) synthesis, which is an essential component of the bacterial cell wall^51^. PG-layer synthesis involves transpeptidase (TP) enzymes, including D, D-TP and L, D-TP. These TP enzymes are the targets of many antibiotic β-lactams as inhibiting their normal functions can lead to cell death by destablizing the cell walls^51^. Although these antibiotics are often inactivated by ARGs, calcium is hardly influenced, which can hinder the binding of L, D-TP to PG-stem by inducing the formation of L, D-TP dimers^51^. Therefore, since the synthesis of ARGs consumes energy but fails to promote viability, a higher concentration of Ca can decrease the relative fitness of the antibiotic resistant bacteria in the environment compared to non-resistant bacteria. Based on such mechanisms, a higher calcium concentration in the water bodies is likely to decrease the overall bacterial quantities regardless of the ARGs carried, and lead to lower concentrations of ARGs detected.

This finding, combined with previous research about Mg, reveals the possibility of developing Group 2A light metals as reagents to control ARB. For example, as one of the major elements in nature, calcium salts are of low toxicity and eco-friendly. Calcium-based reagents might be capable of suppressing antibiotic resistant bacteria in natural environments if added to natural surface water (e.g., exposure recreational water).

However, limitations exist in this study. It is worth noting that overall antibiotic levels in the Yangtze Delta were observed to vary in accordance to seasons, with the number of detected antibiotics and their detection frequencies being significantly lower in summer than in winter^23,24,52,53^, except for tetracyclines^53^. The high flow conditions in summer might dilute the concentration of antibiotics in the surface water^54^, and the higher activity of microorganism and strong sunlight of summer could also result in faster degradation of antibiotics^53,55^. Considering the reported seasonal variance, the findings on antibiotic concentrations can only be generalizable to surface water of the Yangtze River Mouth in summer. Also, impacted by the outbreak of COVID-19 Delta strain in China in the summer of 2021, the sampling phase of this study was emergently terminated, ahead of schedule. The unexpected termination led to a small sample size (15 samples). The small sample size decreases statistical power and increases potential error in the results. Finally, ARG concentrations were of large standard deviations in all four subregions. Though this could be explained as indication of point sources of ARG pollution, one alternative explanation is there was degradation of DNA during the experiments.

## Conclusion

This study detected a high abundance of ARGs in surface water samples collected from the Yangtze River Mouth. Amoxicillin, P, Cr, Mn, Ca, and Sr, assessed through the nonlinear association model, were identified as potential key contributors of ARGs in the Yangtze Delta. Reduction of ARG abundance could be realized through decreasing the concentration of phosphorus in surface water. In addition, Ca was identified to have a potential ARG suppression effect, though future studies are still needed to further evaluate this association.

### Supplementary Information

Supplementary Information 1.

Supplementary Information 2.

## Data Availability

All data generated or analysed during this study are included in this published article and its Supplementary Information files.

## Abbreviations

AMR: Antimicrobial resistance
ARG: Antibiotic resistance gene
