# Supplementary material for: Assessing the nonlinear association of environmental factors with antibiotic resistance genes (ARGs) in the Yangtze River Mouth, China
Source: Sci Rep. 2023 Nov 21;13:20367. doi: 10.1038/s41598-023-45973-9 (PMC10663556; doi:10.1038/s41598-023-45973-9)
Supplement: Supplementary file 2 — Supplementary Information 2. [file 41598_2023_45973_MOESM2_ESM.docx]

**Assessing the Nonlinear Association of Environmental Factors with Antibiotic Resistance Genes (ARGs) in the Yangtze River Mouth, China**

Jiazheng Miao^1, 2^, Yikai Ling^1, 3^, Xiaoyuan Chen^1, 4^, Siyuan Wu^1, 5^, Xinyue Liu^1^, Shixin Xu^1, 6^, Sajid Umar^6^, Benjamin D. Anderson^1, 6, 7,^ *

^1^ Division of Natural and Applied Science, Duke Kunshan University, Kunshan, Jiangsu, China

^2^ Department of Biomedical Informatics, Harvard Medical School, Boston, Massachusetts, United States

^3^ Department of Epidemiology, Bloomberg School of Public Health, Johns Hopkins University, Baltimore, Maryland, United States

^4^ Department of Biomedical Engineering, Whiting School of Engineering, Johns Hopkins University, Baltimore, Maryland, United States

^5^ Department of Statistics, University of Michigan, Ann Arbor, Michigan, United States

^6^ Global Health Research Center, Duke Kunshan University, Kunshan, Jiangsu, China

^7^ Department of Environmental and Global Health, College of Public Health and Health Professions, and Emerging Pathogens Institute, University of Florida, Gainesville, Florida, United States

***Corresponding Authors:**

**Benjamin D. Anderson, MPH, PhD, CPH**

Assistant Professor

Department of Environmental and Global Health, College of Public Health and Health Professions, and Emerging Pathogens Institute, University of Florida, Gainesville, Florida, 32610 United States

Email: [ander88@ufl.edu](mailto:benjamin.anderson2@dukekunshan.edu.cn)

| **Table S1:** Sequences and cycling condition of quantitative PCR experiments of ARG primers. | | | | | |
| --- | --- | --- | --- | --- | --- |
|  | **Orientation** | **Sequence** | **Length** | **Annealing Temperature** | **References** |
| *tetq* | F | AGAATCTGCTGTTTGCCAGTG | 169bp | 55 | [1] |
|  | R | CGGAGTGTCAATGATATTGCA |  |  |  |
| *sul3* | F | TCCGTTCAGCGAATTGGTGCAG | 128bp | 60 | [2] |
|  | R | TTCGTTCACGCCTTACACCAGC |  |  |  |
| *ermB* | F | GGCATTTAACGACGAAACTGGC | 236bp | 57 | [2] |
|  | R | CGCATGGCTTTCAAAAACCAC |  |  |  |
| *ampr* | F | GAGTTTTCGTTCCACTGAGCGTC | 274bp | 60 | [3] |
|  | R | TTAGCAGAGCGAGGTATGTAGGCG |  |  |  |
| *tetA* | F | GCTACATCCTGCTTGCCTTC | 210bp | 55 | [4] |
|  | R | CATAGATCGCCGTGAAGAGG |  |  |  |
| *tetC* | F | CTTGAGAGCCTTCAACCCAG | 418bp | 55 | [4] |
|  | R | ATGGTCGTCATCTACCTGCC |  |  |  |
| *tetE* | F | GTTATTACGGGAGTTTGTTGG | 278bp | 55 | [4] |
|  | R | AATACAACACCCACACTACGC |  |  |  |
| *tetG* | F | GCTCGGTGGTATCTCTGCTC | 468bp | 55 | [4] |
|  | R | AGCAACAGAATCGGGAACAC |  |  |  |
| *tetK* | F | CGAAAACAGACTCGCCAATC | 169bp | 55 | [4] |
|  | R | TCCATAATGAGGTGGGGC |  |  |  |
| *tetL* | F | TCGTTAGCGTGCTGTCATTC | 267bp | 55 | [4] |
|  | R | GTATCCCACCAATGTAGCCG |  |  |  |
| *tetAP* | F | CTTGGATTGCGGAAGAAGAG | 676bp | 55 | [4] |
|  | R | ATATGCCCATTTAACCACGC |  |  |  |
| *tetS* | F | CATAGACAAGCCGTTGACC | 667bp | 55 | [4] |
|  | R | ATGTTTTTGGAACGCCAGAG |  |  |  |
| *tetM* | F | ACAGAAAGCTTATTATATAAC | 171bp | 45 | [5] |
|  | R | TGGCGTGTCTATGATGTTCAC |  |  |  |
| *tetO* | F | ACGGARAGTTTATTGTATACC | 171bp | 45 | [5] |
|  | R | TGGCGTATCTATAATGTTGAC |  |  |  |
| *tetT* | F | AAGGTTTATTATATAAAAGTG | 169bp | 40 | [5] |
|  | R | AGGTGTATCTATGATATTTAC |  |  |  |
| *tetW* | F | GAGAGCCTGCTATATGCCAGC | 168bp | 60 | [5] |
|  | R | GGGCGTATCCACAATGTTAAC |  |  |  |
| *tetBP* | F | AAAACTTATTATATTATAGTG | 169bp | 40 | [5] |
|  | R | TGGAGTATCAATAATATTCAC |  |  |  |
| *sulI* | F | CGCACCGGAAACATCGCTGCAC | 163bp | 56 | [6] |
|  | R | TGAAGTTCCGCCGCAAGGCTCG |  |  |  |
| *sulII* | F | TCCGGTGGAGGCCGGTATATGG | 191bp | 61 | [6] |
|  | R | CGGGAATGCCATCTGCCTTGAG |  |  |  |
| *ereA* | F | AACACCCTGAACCCAAGGGACG | 420bp | 52 | [7] |
|  | R | CTTCACATCCGGATTCGCTCG |  |  |  |
| *ereB* | F | AGAAATGGAGGTTCATACTTACCA | 546bp | 52 | [7] |
|  | R | CATATAATCATCACCAATGGCA |  |  |  |
| *mphA* | F | AACTGTACGCACTTGC | 837bp | 52 | [7] |
|  | R | GGTACTCTTCGTTACC |  |  |  |
| *bla_CTX_M_* | F | ATGTGCAGYACCAGTAARGT | 593bp | 50 | [8] |
|  | R | TGGGTRAARTARGTSACCAGA |  |  |  |
| *bla_TEM_* | F | KACAATAACCCTGRTAAATGC | 936bp | 58 | [8] |
|  | R | AGTATATATGAGTAAACTTGG |  |  |  |
| *bla_SHV_* | F | TTTATCGGCCYTCACTCAAGG | 930bp | 58 | [8] |
|  | R | GCTGCGGGCCGGATAACG |  |  |  |
| *bla_ampC_* | F | CCCCGCTTATAGAGCAACAA | 634bp | 58 | [8] |
|  | R | TCAATGGTCGACTTCACACC |  |  |  |

| **Table S2:** Cross-reactivity of the tetracycline ELISA assay. | |
| --- | --- |
| **Antibiotics** | **Reactivity** |
| tetracycline | 1 |
| minocycline | 0.85 |
| rolitetracycline | 0.7 |
| chlortetracycline | 0.5 |
| demeclocycline | 0.42 |
| oxytetracycline | 0.3 |
| doxycycline | 0.25 |

**Figure S1**: Relative abundance of 22 ARGs detected in screening step. (a) The sum of relative abundance of ARGs. (b) The prevalence of ARGs in the samples. Negative results are represented as grey blocks.


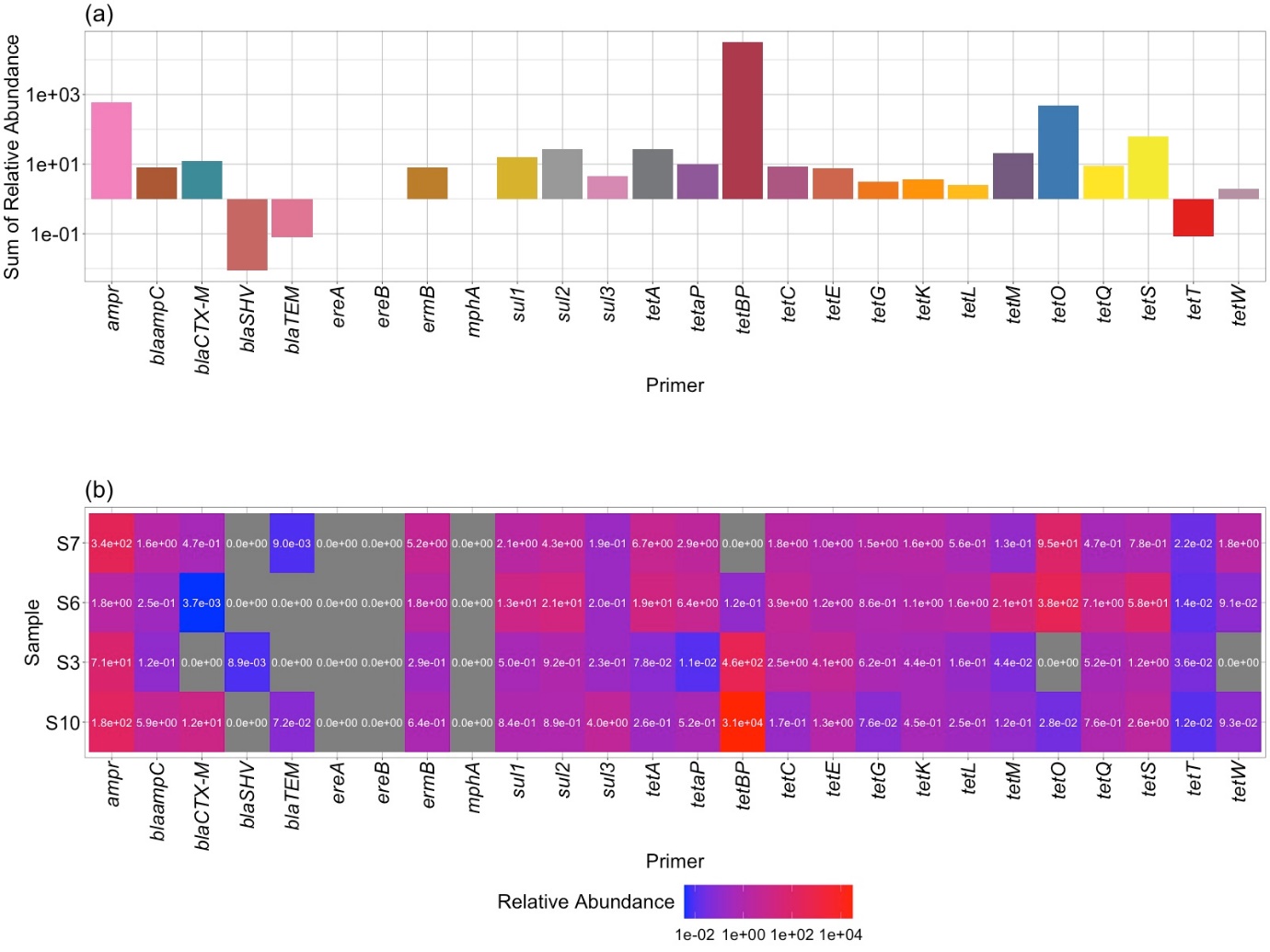


**Figure S2:** Comparison of total tetracycline concentration with previous studies


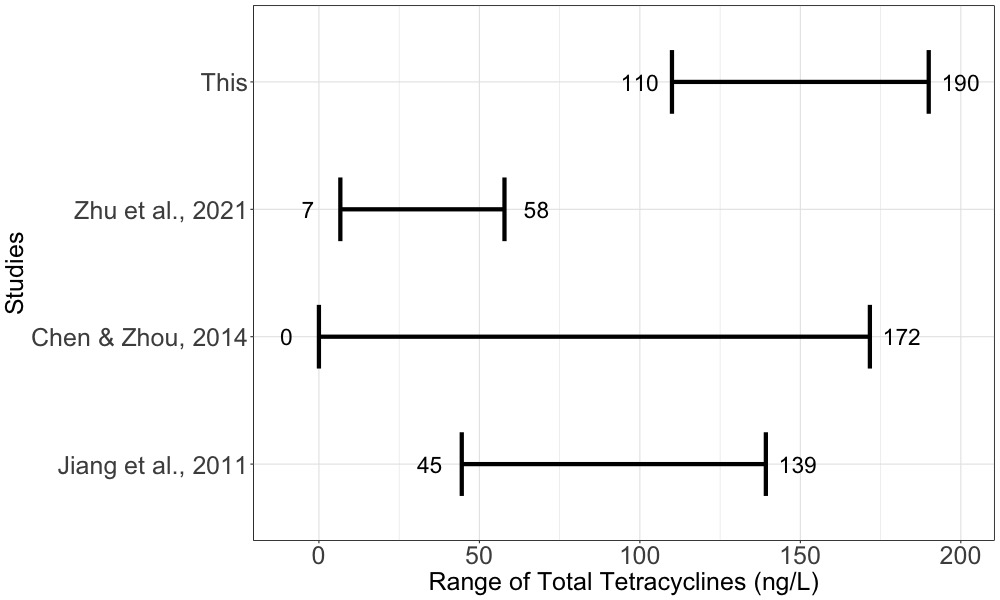


**References**

1. Chen J, Li J, Zhang H, Shi W, Liu Y. Bacterial Heavy-Metal and Antibiotic Resistance Genes in a Copper Tailing Dam Area in Northern China. Frontiers in Microbiology **2019**; 10.

2. Mu Q, Li J, Sun Y, Mao D, Wang Q, Luo Y. Occurrence of sulfonamide-, tetracycline-, plasmid-mediated quinolone- and macrolide-resistance genes in livestock feedlots in Northern China. Environ Sci Pollut Res Int **2015**; 22(9): 6932-40.

3. Wang Q, Hamilton PB, Kang F, Zhu X, Zhang Y, Zhao H. Regional-scale investigation for microbial competition-through-environment interactions modulating antibiotic resistance. Sci Total Environ **2020**; 734: 139341.

4. Ng LK, Martin I, Alfa M, Mulvey M. Multiplex PCR for the detection of tetracycline resistant genes. Mol Cell Probes **2001**; 15(4): 209-15.

5. Aminov RI, Garrigues-Jeanjean N, Mackie RI. Molecular ecology of tetracycline resistance: development and validation of primers for detection of tetracycline resistance genes encoding ribosomal protection proteins. Appl Environ Microbiol **2001**; 67(1): 22-32.

6. Pei R, Kim SC, Carlson KH, Pruden A. Effect of river landscape on the sediment concentrations of antibiotics and corresponding antibiotic resistance genes (ARG). Water Res **2006**; 40(12): 2427-35.

7. Sutcliffe J, Grebe T, Tait-Kamradt A, Wondrack L. Detection of erythromycin-resistant determinants by PCR. Antimicrob Agents Chemother **1996**; 40(11): 2562-6.

8. Shahid M. Citrobacter spp. simultaneously harboring blaCTX-M, blaTEM, blaSHV, blaampC, and insertion sequences IS26 and orf513: an evolutionary phenomenon of recent concern for antibiotic resistance. J Clin Microbiol **2010**; 48(5): 1833-8.
